# Impact of ischemic preconditioning on surgical treatment of brain tumors: a single-center, randomized, double-blind, controlled trial

**DOI:** 10.1186/s12916-017-0898-1

**Published:** 2017-07-25

**Authors:** Arthur H. A. Sales, Melanie Barz, Stefanie Bette, Benedikt Wiestler, Yu-Mi Ryang, Bernhard Meyer, Martin Bretschneider, Florian Ringel, Jens Gempt

**Affiliations:** 1 Department of Neurosurgery, Klinikum rechts der Isar, Technical University of Munich, Ismaninger Str. 22, 81675 Munich, Germany; 2 Department of Neuroradiology, Klinikum rechts der Isar, Technical University of Munich, Ismaninger Str. 22, 81675 Munich, Germany; 3 Department of Anesthesiology, Klinikum rechts der Isar, Technical University of Munich, Ismaninger Str. 22, 81675 Munich, Germany; 4 Department of Neurosurgery, Universitätsmedizin Mainz, Langenbeckstr. 1, 55131 Mainz, Germany

**Keywords:** Ischemic preconditioning, Brain tumor, Glioma, Brain metastasis, Neurooncology, Neurosurgery, Stroke

## Abstract

**Background:**

Postoperative ischemia is a frequent phenomenon in patients with brain tumors and is associated with postoperative neurological deficits and impaired overall survival. Particularly in the field of cardiac and vascular surgery, the application of a brief ischemic stimulus not only in the target organ but also in remote tissues can prevent subsequent ischemic damage. We hypothesized that remote ischemic preconditioning (rIPC) in patients with brain tumors undergoing elective surgical resection reduces the incidence of postoperative ischemic tissue damage and its consequences.

**Methods:**

Sixty patients were randomly assigned to two groups, with 1:1 allocation, stratified by tumor type (glioma or metastasis) and previous treatment with radiotherapy. rIPC was induced by inflating a blood pressure cuff placed on the upper arm three times for 5 min at 200 mmHg in the treatment group after induction of anesthesia. Between the cycles, the blood pressure cuff was released to allow reperfusion. In the control group no preconditioning was performed. Early postoperative magnetic resonance images (within 72 h after surgery) were evaluated by a neuroradiologist blinded to randomization for the presence of ischemia and its volume.

**Results:**

Fifty-eight of the 60 patients were assessed for occurrence of postoperative ischemia. Of these 58 patients, 44 had new postoperative ischemic lesions. The incidence of new postoperative ischemic lesions was significantly higher in the control group (27/31) than in the rIPC group (17/27) (*p* = 0.03). The median infarct volume was 0.36 cm^3^ (interquartile range (IR): 0.0–2.35) in the rIPC group compared with 1.30 cm^3^ (IR: 0.29–3.66) in the control group (*p* = 0.09).

**Conclusions:**

Application of rIPC was associated with reduced incidence of postoperative ischemic tissue damage in patients undergoing elective brain tumor surgery. This is the first study indicating a benefit of rIPC in brain tumor surgery.

**Trial registration:**

German Clinical Trials Register, DRKS00010409. Retrospectively registered on 13 October 2016.

## Background

Remote ischemic preconditioning (rIPC) is the process by which a brief ischemic stimulus applied in a remote tissue protects vital organs (e.g., brain, heart) against subsequent ischemia [1–14].

Some studies have proven the clinical benefits of rIPC in patients undergoing coronary artery bypass surgery [2, 10]. A randomized controlled trial with 57 patients observed a significantly reduced overall serum troponin release after surgery in the rIPC group [2]. In addition, a single-center randomized trial with 329 patients demonstrated a lower geometric mean area under the curve (AUC) for perioperative serum concentrations of cardiac troponin I in the rIPC group [10].

Emerging data from clinical trials have shown that rIPC may also provide neuroprotection. A prospective randomized study involving 68 patients with symptomatic atherosclerotic intracranial arterial stenosis (IAS) evaluated the impact of bilateral arm ischemic preconditioning (BAIPC) on stroke recurrence. The intervention was performed semidaily for 300 days, and the result showed a reduction in stroke incidence from 26.7% in the control group to 7.9% in the BAIPC group at the end of the study [7]. On the other hand, a prospective, randomized, double-blind controlled trial with 180 patients undergoing cardiac surgery with cardiopulmonary bypass failed to demonstrate the efficacy of rIPC in reducing the incidence of postoperative neurocognitive dysfunction [8].

In a phase I study of safety and feasibility, rIPC was shown to be safe and was well tolerated by patients with subarachnoid hemorrhage [5].

The incidence of ischemic tissue damage following resection of gliomas and metastases has been shown to be significant in previous studies and is associated with the occurrence of new postoperative neurological deficits [15–17]. Previous studies have identified postoperative ischemic lesions in 31% of patients with newly diagnosed gliomas, 80% of patients with recurrent gliomas, and 36.1% of patients with metastases who underwent surgical resection [15–17]. Furthermore, a significant impact of infarct volume on overall survival of glioblastoma patients was observed [18]. The prevention of perioperative infarctions is desirable.

We hypothesized that rIPC in patients with intra-axial brain tumors undergoing surgical resection reduces the incidence of postoperative ischemic tissue damage and its sequelae.

## Methods

### Trial design

We conducted a single-center, randomized, parallel, two-group, double-blind, controlled trial. Patients were randomly assigned to two groups, with 1:1 allocation, stratified by tumor type (glioma or metastasis) and previous treatment with radiotherapy.

### Participants and study settings

Eligible patients were adults older than 18 years with suspected primary or metastatic brain tumor planned for elective brain surgery in a tertiary health center (Klinikum rechts der Isar, Munich). Patients younger than 18 years, those with a history of diabetes mellitus (DM), use of oral antidiabetic drugs (OADs), or peripheral artery disease (PAD), pregnant patients, and those who had the operation on an emergency basis without adequate preoperative diagnostic workup were excluded.

### Intervention

The interventions took place in an ancillary room (induction room) after induction of anesthesia prior to surgery. For induction of rIPC, a manual appropriately sized blood pressure cuff was placed on the upper arm and inflated three times for 5 min at 200 mmHg. Between the cycles, the blood pressure cuff was deflated for 5 min to allow reperfusion. In the control group, the blood pressure cuff was placed on the arm and no intervention was performed.

The anesthetic procedures corresponded to the standard procedures for brain tumor surgery. Induction and maintenance of anesthesia were performed via infusion of propofol and remifentanil (total intravenous anesthesia). Mannitol at a dose of 20 g was given for brain relaxation. No specific protocol regarding the use of vasopressors and/or fluid administration was used.

### Outcomes

Early postoperative magnetic resonance (MR) images (within 72 h after surgery) were evaluated for occurrence of ischemic lesions (primary endpoint) and ischemic lesion volumes (secondary endpoint).

**Fig. 1:**
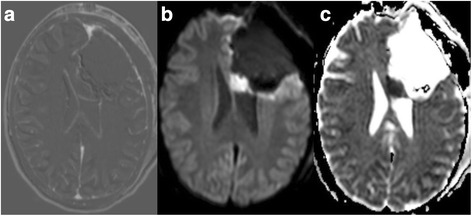
**a** shows a postoperative subtraction, **b** a postoperative diffusion-weighted image (*DWI*, b 1000), and **c** the corresponding apparent diffusion coefficient (*ADC*) map. Images **a**–**c** show an example of a postoperative ischemia with restricted diffusion in the genu of the corpus callosum in a patient diagnosed with an anaplastic oligodendroglioma

Focal hyperintensity on diffusion-weighted images (DWIs) and a corresponding hypointensity on apparent diffusion coefficient (ADC) maps were the morphological criteria used to define ischemic lesions (Fig. 1). We excluded areas of restricted diffusion related to methemoglobin [17]. A neuroradiologist blinded to treatment allocation and clinical course evaluated the imaging studies.

Magnetic resonance imaging (MRI) studies were performed with a whole-body 3-T imaging system (Achieva 3 T, Philips Electronics N.V.) using an 8-/16-channel head coil. ADC maps and DWIs were included in this study. DWIs were obtained through single-shot echo planar imaging with 2 b values of 0 and 1000 s/mm^2^. Isotropic DWIs and ADC maps were calculated automatically with the following parameters: repetition time (TR) 3388 or 8413 ms, echo time (TE) 55 ms; image resolution 2 × 2 × 2 mm or 1.6 × 1.8 × 5 mm. T2-weighted fluid-attenuated inversion recovery (FLAIR: TR 12,000 ms, TE 140 ms, inversion time 2850 ms), a T2-weighted gradient echo (TR 813 ms, TE 16 ms), and a T1-weighted spin echo (TR 494 ms, TE 10 ms) prior to and after intravenous administration of 0.1 mmol/kg of gadopentetate dimeglumine were also acquired.

The treating neurosurgeon assessed the occurrence and severity of new postoperative neurological deficits or worsening of preoperative neurological function before hospital discharge and 3 months after surgery. Motor function was assessed with the Medical Research Council muscle strength grading system. The Karnofsky Performance Status Scale (KPS) was used to measure functional status.

### Sample size

Sample size determination was difficult due to the lack of previous studies investigating the impact of rIPC on occurrence of perioperative ischemic lesions. Based on a randomized trial published in 2012 [7], we hypothesized a reduction in incidence of new ischemic events greater than 50% in the rIPC group (from 60% to 23%). Considering a two-sided test with an alpha of 0.05 and statistical power of 80%, we estimated that 24 patients would be required for each group. Additional patients were included in each group considering the possible dropout and inequality in patient allocation. Therefore, 30 patients per group were planned.

### Randomization and blinding

A computer-generated list of random numbers was created for assignment of participants to either the rIPC group or the control group with a 1:1 allocation using random block sizes of 6, 8, and 10 stratified according to previous radiotherapy and tumor type (brain metastasis vs. glioma). A researcher who was not involved in treatment and outcome assessment generated the random allocation sequence and assigned participants to interventions (BW). AHAS enrolled the participants and conducted the interventions. Only the investigator responsible for assigning patients to interventions (BW) had access to the random allocation sequence.

Patients and outcome assessors were blinded to treatment allocation (double-blind study). In addition, the neurosurgeons remained blinded, since interventions were conducted in the induction room before surgery. Anesthetists left the ancillary room while the interventions were performed.

### Statistical analysis

A descriptive data analysis, Pearson chi-square test, Student’s *t* test, Fisher’s exact test, and Mann-Whitney U test were performed using IBM SPSS Statistics version 23.0. Data are presented as mean (standard deviation), median (interquartile range), or number of patients. Treatment groups were compared for the primary outcome (incidence of new ischemic lesions) using the Pearson chi-square test (two-sided). Due to our small sample size, the infarct volume data did not follow a normal distribution. Therefore, we performed the Mann-Whitney U test (two-sided) to compare the two treatment groups. The relative risk (RR) and Pearson correlation coefficient (*r*) were measured in order to quantify effect sizes. A *p* value of less than 0.05 was considered statistically significant.

## Results

**Fig. 2:**
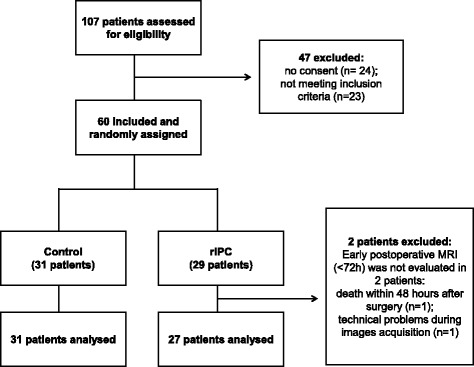
Flowchart of the trial profile. One hundred seven patients were assessed for eligibility, of whom 60 were included and randomly assigned to one of two treatment groups (29 patients in the rIPC group and 31 patients in the control group). Two patients were excluded after randomization: early postoperative MRI was not evaluated in 2 patients in the rIPC group. Therefore, 58 patients were assessed for occurrence of postoperative ischemia

Between September 2015 and June 2016, 107 patients with suspected primary or metastatic brain tumors were assessed for eligibility, of whom 60 patients were included and randomly assigned to the rIPC group (29 patients) or the control group (31 patients). Early postoperative MRI was not evaluated in one patient in the rIPC group due to technical problems during image acquisition. Another patient in the rIPC group had died within 48 h after surgery due to clinical complications and severe comorbidities. Therefore, only 58 of the 60 patients were assessed for occurrence of postoperative ischemia. Figure 2 shows the trial profile.

The participants were followed from September 2015 until September 2016 for evaluation of the occurrence of permanent neurological deficits.

### Descriptive data analysis

Twenty-nine patients were male and 29 were female. The mean age at time of surgery was 56.6 ± 13.7 years (range: 32–80). Of the 58 patients, 35 had a primary brain tumor and 23 had a metastatic brain tumor. Among patients with primary brain tumors, 7 patients had a low-grade glioma (LGG) (World Health Organization (WHO) grade I in 1 case, WHO grade II in 6), and 28 patients a high-grade glioma (HGG) (WHO grade III in 15 cases, WHO grade IV in 13). Twelve patients had a glioblastoma, 9 an anaplastic astrocytoma, 5 a diffuse astrocytoma, 5 an anaplastic oligodendroglioma, 1 an oligodendroglioma, 1 an anaplastic oligoastrocytoma, 1 a ganglioglioma, and 1 a gliosarcoma. *O*(6)-methylguanine-DNA methyltransferase (MGMT) methylation was found in 12 patients, whereas isocitrate dehydrogenase 1 (IDH1) mutation was detected in 17 patients and 1p/19q codeletion in 8 patients.

Adenocarcinoma was the most common histological type among patients with metastatic brain tumors, affecting 10 patients, followed by melanoma (4 patients), undifferentiated carcinoma (2 patients), squamous cell carcinoma (2 patients), and other subtypes (5 patients).

The primary sites in patients with metastatic tumors were as follows: lung cancer in 10 cases, melanoma in 4, upper gastrointestinal tract tumors in 2, ovarian cancer in 1, urinary tract cancer in 1, and unknown in 5 cases.

Seventeen patients had had previous treatment with radiotherapy, whereas 20 patients had received chemotherapy prior to surgery. Among the patients with primary brain tumors who had received chemotherapy prior to surgery, 10 were treated with temozolomide, whereas only one patient had received lomustine (CCNU).

The main tumor location was frontal in 30 cases, temporal in 15, and parietal in 5, in the basal ganglia in 3 cases, and in other locations in 5. Twenty-one patients had left-sided tumors, 26 right-sided tumors, and 11 bilateral tumors.

Fifty-six of the 58 surgical procedures were performed by eight board-certified neurosurgeons. In detail, senior surgeons with a mean experience of 17.5 years (range 14–25 years) performed 43 surgeries, while surgeons with an intermediate experience level (8.5 years, range 7–10 years) performed 13 surgeries. Two of the 58 surgical procedures were performed by chief residents under supervision of one of the above-mentioned board-certified neurosurgeons.

The mean duration of surgery was 2.71 ± 0.87 h in the rIPC group and 2.62 ± 0.9 h in the control group. Forty-four patients were classified as American Society of Anesthesiologists Physical Status (ASA PS) 1 or 2 (low risk), and 9 as ASA PS 3 (intermediate risk). An ASA PS classification was not available for 5 patients. The use of intraoperative neurophysiological monitoring was similar in both groups (20 patients in the rIPC group vs. 19 patients in the control group). Gross total resection was achieved in 26 patients, near total resection (≥90% but <100%) in 21, and subtotal resection in 11.

The baseline characteristics did not differ between treatment groups (Table 1).

**Table 1:** Patient characteristics

		rIPC group (*n* = 27)	Control (*n* = 31)
General data	Age (years)	58.89 (±13.5)	54.77 (±13.9)
	Sex (male/female)	12/15	17/14
	BMI	25.73 (±6.18)^a^	25.42 (±4.12)^b^
Previous medical conditions	Arterial hypertension	6	10
	Coronary artery disease	2	3
	Hypothyroidism	6	3
	Atrial fibrillation	1	0
	Hypercholesterolemia	0	4
	Previous stroke	0	0
	Smokers	3	5
	Ex-smokers	0	2
Regular medications	Aspirin	2	3
	Beta blockers	4	2
	Calcium channel blockers	3	1
	ACE inhibitors	5	6
	Anticoagulants	1	0
	Anticonvulsants	10	14
	Diuretics	4	3
	Statins	3	2
	Levothyroxine	6	3
	Antidepressants	3	4
	Other drugs	1	5
Clinical data	Patients undergoing first resection	10	15
	Previous radiotherapy	8	9
	Previous chemotherapy	10	10
	Glioma patients previously treated with temozolomide	6	4
	Glioma patients previously treated with CCNU	0	1
	Preoperative Karnofsky (%)	90 (80–100)	100 (80–100)
Tumor location	Frontal	15	15
	Temporal	6	9
	Parietal	2	3
	Basal ganglia	1	2
	Other locations	3	2
	Left hemisphere	10	11
	Right hemisphere	12	14
	Bilateral tumors	5	6
Surgical data	ASA PS 1	1	3
	ASA PS 2	17	23
	ASA PS 3	6	3
	Surgery duration (h)	2.71 (±0.87)	2.62 (±0.9)
	Use of intraoperative neuromonitoring (MEP/SEP)	20	19
	Gross total resection	13	13
	Near total resection	9	12
	Subtotal resection	5	6
	Intraoperative blood loss (ml)	300 (200–300)^c^	300 (200–600)^d^
	Hypoxemia (SaO_2_ ≤ 92%)	1 ^e^	0 ^f^
	Hypotension (MAP ≤65 mmHg)	1	4
	Use of intraoperative corticosteroids	0	0
	Intraoperative vessel damage	0	0
Histopathological findings in patients with glioma	LGG (WHO I and II)	3	4
	HGG (WHO III and IV)	13	15
	Glioblastoma	6	6
	Gliosarcoma	0	1
	Diffuse astrocytoma	2	3
	Anaplastic astrocytoma	4	5
	Oligodendroglioma	0	1
	Anaplastic oligodendroglioma	2	3
	Anaplastic oligoastrocytoma	1	0
	Ganglioglioma	1	0
	MGMT methylation	5	7
	1p/19q codeletion	4	4
	IDH1 mutation	7	10
Histopathological findings in patients with metastasis	Adenocarcinoma	6	4
	Undifferentiated carcinoma	0	2
	Melanoma	3	1
	Squamous cell carcinoma	1	1
	Other	1	4

Data are presented as mean (standard deviation (*SD*)), median (interquartile range (*IR*)), or number of patients. *BMI* body mass index, *ACE* angiotensin-converting enzyme, *CCNU* lomustine, *ASA PS* American Society of Anaesthesiologists Physical Status classification, *MEP/SEP* motor- and somatosensory-evoked potential monitoring, *MAP* mean arterial pressure, *LGG* low-grade glioma, HGG high-grade glioma, *WHO* World Health Organization, *MGMT O*(6)-methylguanine-DNA methyltransferase, *IDH1* isocitrate dehydrogenase 1

^a^Data obtained from 16 patients

^b^Data obtained from 20 patients

^c^Data obtained from 21 patients

^d^Data obtained from 27 patients

^e^Data obtained from 24 patients

^f^Data obtained from 29 patients

### Ischemic preconditioning and postoperative ischemic tissue damage

**Fig. 3:**
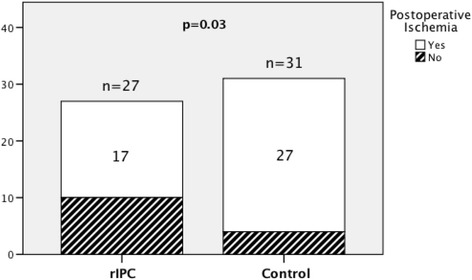
Ischemic preconditioning and postoperative ischemic lesions: the bar graph shows the incidence of new ischemic lesions in both treatment groups. The incidence of postoperative ischemic lesions was significantly higher in the control group (27/31) than in the rIPC group (17/27). Pearson chi-square test, *p* = 0.03

Forty-four of 58 patients had new postoperative ischemic lesions. The incidence of new postoperative ischemic lesions was significantly higher in the control group (27/31) than in the rIPC group (17/27) (Pearson chi-square test, *p* = 0.03; RR = 0.722, 95% confidence interval (CI) 0.525–0.994). See Table 2 and Fig. 3.

**Table 2:** Remote ischemic preconditioning: outcomes

Outcomes	rIPC (*n* = 27)	Control (*n* = 31)	*p* value	RR (CI 95%)	Absolute risk reduction	Pearson’s *r* (CI 95%)	NNT
Postoperative ischemia	17	27	0.03	0.722 (0.525–0.994)	24.1%	NA	4.1
Median infarct volume (cm^3^)	0.36 (0.0–2.35)	1.30 (0.29–3.66)	0.09	NA	NA	0.21 (-0.03–0.46)	NA
New neurological deficits	4	5	1	0.918 (0.274–3.078)	NA	NA	NA
Worsening of preoperative deficits	3	3	1	1.148 (0.252–5.222)	NA	NA	NA

Data are presented as median (interquartile range) or number of patients

*RR* relative risk, *CI 95%* 95% confidence interval, *NNT* number needed to treat *NA* not applicable

**Fig. 4:**
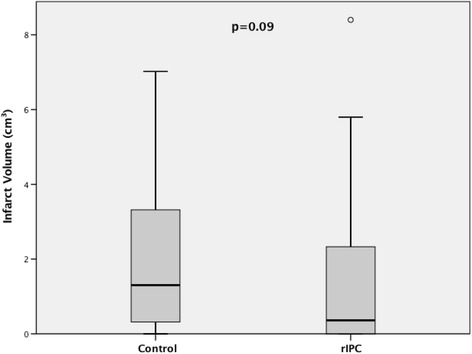
Ischemic preconditioning and infarct volume: the boxplot shows the median infarct volume in both treatment groups. The median infarct volume was 0.36 cm^3^ (IR: 0.0–2.35) in the rIPC group compared with 1.30 cm^3^ (IR: 0.29–3.66) in the control group. Mann-Whitney U test, *p* = 0.09

Although we observed a clear trend, the association between ischemic preconditioning and infarct volumes was not significant. The median infarct volume was 0.36 cm^3^ (IR: 0.0–2.35) in the rIPC group compared with 1.30 cm^3^ (IR: 0.29–3.66) in the control group (Mann-Whitney U test, *p* = 0.09). See Fig. 4.

### Ischemic preconditioning and neurological deficits

New neurological deficits occurred in 4 of 27 patients in the rIPC group: anomic aphasia in 1, severe motor deficit (muscle strength (MS): 0–2/5) in 1, mild to moderate motor deficit (MS: 3–4/5) in 2 cases, and dysphagia in 1 case. The deficits were permanent in 2 of these patients at 3 months follow-up (anomic aphasia in 1, severe motor deficit in another). One patient presented with recovery of neurological function, and 1 patient died within 1 month after surgery.

In the control group, new neurological deficits were found in 5 of 31 patients: non-fluent aphasia in 1 case, dysarthria in 1 case, sensitive deficit in 2 cases, and mild to moderate motor deficit in 3 cases. Of these 5 patients, one had permanent deficits at 3 months follow-up (anomic aphasia and mild to moderate motor deficit). Three patients have shown improvement in neurological function, and one patient was lost to follow-up.

There was no significant difference between the two groups with respect to incidence of new neurological deficits (Fisher's exact test; *p* = 1).

Three of 27 patients in the rIPC group experienced postoperative deterioration of neurological symptoms: aphasia in 2 cases, severe motor deficit in 2 cases, and mild to moderate motor deficit in 1 case. At 3 months follow-up, only one of these patients had a permanent deficit (mild to moderate motor deficit).

In the control group, three patients had a postoperative worsening of neurological function (severe motor deficit). One of these patients presented a partial improvement of motor function (mild to moderate deficit) at 3 months follow-up. The other two patients were lost to follow-up.

## Discussion

Our study demonstrated that rIPC was associated with a reduced incidence of new postoperative ischemic lesions in patients undergoing elective brain tumor surgery. The benefits of rIPC in patients undergoing cardiac surgery have been shown to be significant in many clinical studies [2, 3, 10]. Myocardial infarction, as measured by a surrogate parameter (serum troponin levels), has been shown to be less severe in patients assigned to the preconditioning group [2, 10]. However, the impact of rIPC on the incidence of postoperative ischemic lesions in patients with brain tumors has not been evaluated to date.

Previous studies have demonstrated that ischemic preconditioning confers protection against cerebral ischemia and its sequelae [7, 19, 20]. Wegener et al. were able to show that patients with transient ischemic attacks (TIAs) prior to stroke had smaller infarct volumes than patients without a history of TIA, and this was associated with milder clinical deficits [19]. A prospective randomized study involving 68 patients with symptomatic atherosclerotic IAS showed a reduction in stroke incidence from 26.7% in the control group to 7.9% in the ischemic preconditioning group at the end of the study [7]. Moreover, Chan et al. evaluated the effects of ischemic preconditioning (IPC) during clipping of cerebral aneurysm. In the IPC group, the proximal artery was briefly occluded for 2 min followed by a 30-min reperfusion. The decline of oxygen tension (PtO_2_) and pH in tissues at risk was delayed in the IPC group compared to the control group [20].

In our study, 44 of 58 patients had new postoperative ischemic lesions. This incidence was similar to that reported in previous studies involving patients with brain tumors [15–17].

The primary outcome of our study was the incidence of new ischemic lesions. We found an absolute risk reduction of 24% and a number needed to treat (NNT) of 4.1, which indicates that rIPC is effective in reducing the incidence of postoperative ischemic changes. Our sample size was determined to evaluate this endpoint, which proved to be significant but too small to determine whether the association between rIPC and infarct volume is significant as well. In addition, infarct volumes were generally small in both groups, which is consistent with the results of previous studies [16, 18]. However, we found a trend toward smaller infarct volumes in the rIPC group. Further randomized trials with larger sample sizes are necessary to investigate this association.

A limitation of our study is that patients were not evaluated separately according to underlying disease (glioma or brain metastasis). Although both are space-occupying brain lesions, the pathological features vary considerably, and this may impact surgical resection and occurrence of postoperative complications, including ischemic events. Glial tumors infiltrate the surrounding tissue in contrast to brain metastases, which are usually well circumscribed [21, 22]. Consequently, surgical resection of brain metastases is often considered easier and less damaging to the surrounding brain tissue than the resection of glial tumors [22]. Previous studies have demonstrated differences in incidence of new postoperative ischemic lesions between these two entities [16, 17]. A retrospective study involving 122 patients with brain metastases showed that 44 patients (36.1%) had postoperative ischemic lesions, whereas another retrospective study involving glioma patients showed an incidence of 31% (26 of 84 patients) of postoperative ischemic lesions in patients with newly diagnosed gliomas and 80% (20 of 25 patients) in patients with recurrent gliomas [16, 17]. Therefore, in our study, care was taken to balance treatment groups through stratification.

The occurrence of new postoperative neurological deficits and postoperative worsening of neurological function did not differ significantly between treatment groups. Previous retrospective studies have demonstrated a significant association between incidence of ischemic lesions and occurrence of new neurological deficits [16, 17, 23]. A case-control study involving 84 patients who underwent glioma resection (42 patients with postoperative neurological deficits and 42 patients without new acquired deficits) has shown that postoperative ischemic lesions were more often seen in patients with new neurological deficits (63% vs. 44%) [23]. The incidence of new neurological deficits in our sample was similar to those reported in previous studies [16, 17, 24, 25].

Considering that deterioration of neurological function was a secondary outcome in this study, we cannot consider these results as definitive. The sample size was not determined to investigate this outcome and is insufficient to establish or refute this association.

## Conclusions

Application of rIPC was associated with reduced incidence of perioperative ischemic infarctions in patients undergoing elective brain tumor surgery. This is the first study indicating a benefit of rIPC in brain tumor surgery. rIPC may be effective in improving cerebral perfusion in patients undergoing brain tumor resection.

## Abbreviations

ACE: Angiotensin-converting enzyme
ADC: Apparent diffusion coefficient
ASA PS: American Society of Anesthesiologists Physical Status classification System
AUC: Area under the curve
BAIPC: Bilateral arm ischemic preconditioning
BMI: Body mass index
CCNU: Lomustine
DM: Diabetes mellitus
DWI: Diffusion-weighted image
FLAIR: Fluid-attenuated inversion recovery
HGG: High-grade glioma
IAS: Intracranial arterial stenosis
IDH1: Isocitrate dehydrogenase 1
IPC: Ischemic preconditioning
IR: Interquartile range
KPS: Karnofsky Performance Status Scale
LGG: Low-grade glioma
MAP: Mean arterial pressure
MEP/SEP: Motor- and somatosensory-evoked potential monitoring
MGMT: *O*(6)-methylguanine-DNA methyltransferase
MRI: Magnetic resonance imaging
MS: Muscle strength
OAD: Oral antidiabetic drug
PAD: Peripheral artery disease
pH: Potential of hydrogen
PtO_2_: Tissue oxygen tension
rIPC: Remote ischemic preconditioning
SD: Standard deviation
TE: Echo time
TIA: Transient ischemic attack
TR: Repetition time
WHO: World Health Organization

## References

[1] Steiger H-J, Hänggi D (2007). Ischaemic preconditioning of the brain, mechanisms and applications. Acta Neurochir (Wien).

[2] Hausenloy DJ, Mwamure PK, Venugopal V, Harris J, Barnard M, Grundy E (2007). Effect of remote ischaemic preconditioning on myocardial injury in patients undergoing coronary artery bypass graft surgery: a randomised controlled trial. Lancet..

[3] McCrindle BW, Clarizia NA, Khaikin S, Holtby HM, Manlhiot C, Schwartz SM (2014). Remote ischemic preconditioning in children undergoing cardiac surgery with cardiopulmonary bypass: a single-center double-blinded randomized trial. J Am Heart Assoc..

[4] Heusch G, Bøtker HE, Przyklenk K, Redington A, Yellon D (2015). Remote ischemic conditioning. J Am Coll Cardiol..

[5] Koch S, Katsnelson M, Dong C, Perez-Pinzon M (2011). Remote ischemic limb preconditioning after subarachnoid hemorrhage: a phase Ib study of safety and feasibility. Stroke..

[6] Iliodromitis EK, Lazou A, Kremastinos DT (2007). Ischemic preconditioning: protection against myocardial necrosis and apoptosis. Vasc Health Risk Manag..

[7] Meng R, Asmaro K, Meng L, Liu Y, Ma C, Xi C (2012). Upper limb ischemic preconditioning prevents recurrent stroke in intracranial arterial stenosis. Neurology..

[8] Meybohm P, Renner J, Broch O, Caliebe D, Albrecht M, Cremer J (2013). Postoperative neurocognitive dysfunction in patients undergoing cardiac surgery after remote ischemic preconditioning: a double-blind randomized controlled pilot study. Biondi-Zoccai G, editor. PLoS One.

[9] Stenzel-Poore MP, Stevens SL, Xiong Z, Lessov NS, Harrington CA, Mori M (2003). Effect of ischaemic preconditioning on genomic response to cerebral ischaemia: similarity to neuroprotective strategies in hibernation and hypoxia-tolerant states. Lancet..

[10] Thielmann M, Kottenberg E, Kleinbongard P, Wendt D, Gedik N, Pasa S (2013). Cardioprotective and prognostic effects of remote ischaemic preconditioning in patients undergoing coronary artery bypass surgery: a single-centre randomised, double-blind, controlled trial. Lancet..

[11] Zhao Z, Corvera JS, Halkos ME, Kerendi F, Wang N, Guyton RA (2003). Inhibition of myocardial injury by ischemic postconditioning during reperfusion: comparison with ischemic preconditioning. Am J Physiol - Hear Circ Physiol..

[12] Dirnagl U, Becker K, Meisel A (2009). Preconditioning and tolerance against cerebral ischaemia: from experimental strategies to clinical use. Lancet Neurol..

[13] Helgeland E, Breivik LE, Vaudel M, Svendsen OS, Garberg H, Nordrehaug JE (2014). Exploring the human plasma proteome for humoral mediators of remote ischemic preconditioning — a word of caution. PLoS One..

[14] Tulu S, Mulino M, Pinggera D, Luger M, Wurtinger P, Grams A (2015). Remote ischemic preconditioning in the prevention of ischemic brain damage during intracranial aneurysm treatment (RIPAT): study protocol for a randomized controlled trial. Trials..

[15] Gempt J, Krieg SM, Hüttinger S, Buchmann N, Ryang Y-M, Shiban E (2013). Postoperative ischemic changes after glioma resection identified by diffusion-weighted magnetic resonance imaging and their association with intraoperative motor evoked potentials. J Neurosurg..

[16] Gempt J, Gerhardt J, Toth V, Huettinger S, Ryang YM, Wostrack M, Krieg SM (2013). Postoperative ischemic changes following brain metastasis resection as measured by diffusion-weighted magnetic resonance imaging. J Neurosurg..

[17] Gempt J, Förschler A, Buchmann N, Pape H, Ryang Y-M, Krieg SM (2013). Postoperative ischemic changes following resection of newly diagnosed and recurrent gliomas and their clinical relevance. J Neurosurg..

[18] Bette S, Wiestler B, Kaesmacher J, Huber T, Gerhardt J, Barz M (2016). Infarct volume after glioblastoma surgery as an independent prognostic factor. Oncotarget..

[19] Wegener S, Gottschalk B, Jovanovic V, Knab R, Fiebach JB, Schellinger PD (2004). Transient ischemic attacks before ischemic stroke: preconditioning the human brain? A multicenter magnetic resonance imaging study. Stroke..

[20] Chan MT V, Boet R, Ng SCP, Poon WS, Gin T. Effect of ischemic preconditioning on brain tissue gases and pH during temporary cerebral artery occlusion. Acta Neurochir. Suppl. 2005;Suppl 95:93–6. 10.1007/3-211-32318-x_20 16463828

[21] Sunwoo L, Yun TJ, You S-H, Yoo R-E, Kang KM, Choi SH (2016). Differentiation of glioblastoma from brain metastasis: qualitative and quantitative analysis using arterial spin labeling MR imaging. PLoS One..

[22] Lee S, Hwang S, Im SB, Kim B (2016). Surgical resection of non-glial tumors in the motor cortex. Brain Tumor Res Treat..

[23] Jakola AS, Berntsen EM, Christensen P, Gulati S, Unsgård G, Kvistad KA (2014). Surgically acquired deficits and diffusion weighted MRI changes after glioma resection — a matched case-control study with blinded neuroradiological assessment. PLoS One..

[24] Gulati S, Jakola AS, Nerland US, Weber C, Solheim O (2011). The risk of getting worse: Surgically acquired deficits, perioperative complications, and functional outcomes after primary resection of glioblastoma. World Neurosurg..

[25] Ringel F, Pape H, Sabel M, Krex D, Bock HC, Misch M (2016). Clinical benefit from resection of recurrent glioblastomas: Results of a multicenter study including 503 patients with recurrent glioblastomas undergoing surgical resection. Neuro Oncol..

